# Medical Inpatient Management of Hypoglycaemia: Nursing Intervention Quality Improvement Project

**DOI:** 10.7759/cureus.119618

**Published:** 2026-11-13

**Authors:** Dominic Annable

**Affiliations:** 1 General Medicine, Macclesfield District General Hospital, Macclesfield, GBR

**Keywords:** anti-diabetic drugs, document analysis, hypoglycaemia, nursing skills, quality improvement projects, type 1 diabetes mellitus, types 2 diabetes

## Abstract

Background

Hypoglycaemia presents a common and serious complication for patients with diabetes. Hypoglycaemia can also extend the length of hospital stays and increase the risk of in-patient health complications. This quality improvement project assessed and sought to improve compliance with local trust guidance through targeted nursing interventions.

Methods

This single-centre, small study included all medical inpatients with hypoglycaemia of <4.0 mmol/L. Patients within the emergency department and surgical specialities were excluded. The sample size consisted of 20 hypoglycaemic episodes in each group, selected at random from medical wards. In the baseline group, 13 patients were found compared to six in the post-intervention group. The baseline study took place in January 2026, and the post-intervention study took place from June to August 2026. Data were evaluated with a standardised audit proforma and the digital care system to assess glucose levels and nursing documentation. The intervention in June 2026 used one-to-one on-site teaching, feedback, and guidance posters in order to increase compliance. Aspects of nursing documentation completeness, repeat glucose monitoring, treatment pathway choice, escalation to medical staff, and preventative management planning were assessed.

Results

Both cohorts possessed eight (40%) patients with severe hypoglycaemia defined as <3.0 mmol/L in this study. In the post-intervention group, the use of route A treatment (such as oral glucose or fruit juice) had increased by 40% (n=8). There was a statistically significant increase in adherence to trust guidance, given the documented patient’s well-being (OR 11.14, 95% CI 1.11-112.01; p=0.033, φ=0.445). On the contrary, there was a 10% (n=2) increase in no general nursing documentation present to 20% (n=4). Specific patient status not documented remained constant at 30% (n=6), and there was no statistically significant improvement in well-being status documented (OR 1.00, 95% CI 0.26-3.87; p=1.000, φ=0.0). Adherence to repeat glucose targets remained poor; monitoring within 15 minutes dropped from 25% (n=5) to 5% (n=1) post-intervention. Delays (>1 hour) remained common. In the 15-minute interval, there was no statistical improvement between groups (OR 0.18, 95% CI 0.02-1.81; p=0.167, φ=0.264). The medical escalation of hypoglycaemic episodes was reduced from 35% (n=7) in the baseline group to 15% (n=3) in the post-intervention group; preventative management plans decreased from 40% (n=8) to 30% (n=6). The post-intervention group was type 2 diabetes dominant at 95% (n=19), compared to 70% (n=14) in the baseline group; however, statistically, there was no difference between groups (p=0.09, 95% CI 75.479, OR 8.143, φ=0.329). Insulin treatment alone use was also 20% (n=4) higher in the post-intervention group at 60% (n=12) versus 40% (n=8) in the baseline group.

Conclusion

In the cohort of nurses who responded to the feedback questionnaire, 55% (n=11) reported an increase in self-reported confidence. There was a statistically significant increase in the presence of appropriate treatment pathways in the post-intervention group. Lower rates of route C management and intravenous glucose usage were observed in the post-intervention group. However, improvements were not observed in glucose monitoring times, general documentation completeness and specific statements of patients' well-being during hypoglycaemic episodes.

## Introduction

Hypoglycaemia is defined as a plasma glucose level of less than 4 mmol/L according to the National Institute for Health and Care Excellence (NICE). According to the American Diabetes Association (ADA), further classification exists into level 1, defined as glucose <3.9 mmol/L; level 2 as < 3.0 mmol/L; and level 3 involving a severely altered mental or physical state. Hypoglycaemia can present with symptoms of shakiness, confusion, sweating and tachycardia. The National Diabetes Inpatient Audit (NaDIA) reports in 2019 that around one in five diabetic inpatients had at least one hypoglycaemic episode; severe hypoglycaemia occurs less commonly yet with a higher risk of adverse outcomes. Hypoglycaemia has been correlated with higher patient mortality rates and increased length of hospital stay. Complications also include cardiac arrhythmias, seizures, risk of stroke and emotional distress to patients. In addition, the risk of recurrence rises after a first hypoglycaemic episode. According to Diabetes UK, around one in six beds are occupied by patients with diabetes [1-6].

For patients with diabetes, hypoglycaemia can be precipitated by infection, fasting, reduced activity, anti-diabetic medication such as sulphonylurea and insulin dosing or timings. Non-diabetic hypoglycaemia can also occur due to thyroid disease, adrenal insufficiency, renal and hepatic dysfunction and insulinomas [7-9].

For health care professionals, urgent management of hypoglycaemia is driven by local protocol, international standardised procedure and professional judgement. Treatment can range from oral sugar-dense carbohydrates, such as oral glucose gel or juice, to intravenous glucose infusions. In all cases, swift re-monitoring is crucial. Standards and guidance can be found at the NICE, the Association of British Clinical Diabetologists (ABCD) and the ADA [10].

Previous quality improvement projects (QIPs) have shown that targeted interventions can reduce hypoglycaemia rates. Proven strategies include educational posters, introduction of management protocols, regular teaching sessions, direct resident feedback and electronic reminders [11,12]. This study took inspiration from these interventions to help improve hypoglycaemia management within the hospital. Local trust hypoglycaemia guidance concordance had not been repeated in several years; therefore, as part of patient safety, best medical practice, and to initiate further improvement cycles, this study was commenced.

Study aims

The primary outcome was to measure and observe a statistically significant increase in compliance with local trust guidance, defined by the proportion of patients who had the suggested treatment modality administered according to their documented well-being status and glucose level.

Secondary outcomes were to observe a statistically significant increase in the proportion of capillary glucose samples monitored within the suggested 15 minutes.

Thirdly, to measure a statistically significant rise in the proportion of patients who had their well-being status documented fully. This was defined by the presence of terms such as confused, disorientation, seizures, able to swallow or other synonymous terms associated with hypoglycaemia signs, which were classified A to E. The timeframe for the above cycles was set at 12 months. This includes time for planning, orchestrating further cycles and presenting the findings to the local trust.

Escalation to the medical team and data points of prevention planning were observed; however, they were not seen as outcome measures. The presence of a clustering effect and unreported regularity of nurses working with individual patients meant that the result may lack validity; however, these factors were taken into consideration.

## Materials and methods

Design

This QIP used criterion-based sampling. This included adults aged 18 or over who were medical inpatients and had a glucose concentration of less than 4 mmol/L, as defined by the Joint British Diabetes Societies for In-Patient Care (JBDS) [10]. Patients who were pregnant or were from a surgical speciality, maternity ward, paediatrics, or emergency department were excluded from the sample cohort.

From 12 January to 18 February 2026, and then from 12 June to 05 August 2026, all medical in-patients receiving nursing glucose monitoring were inspected in order to detect hypoglycaemia. Cases were retrospectively identified using the Meditech Expanse Digital Clinical System (DCS) electronic patient record system by Medical Information Technology, Inc. (Westwood, Massachusetts, USA). DCS includes time-stamped glucose readings taken by nursing staff, responsive management plans used and the coinciding documentation. Once patients were identified this way, their hypoglycaemia levels were tracked until resolution. Resolution was defined as reaching a glucose of >4.0 mmol/L. Medical wards were allocated a number, then the Random.org website [13] was used to randomise the order in which wards were scanned. We collected data on a paper proforma provided by the trust and then collated them in a Microsoft Excel spreadsheet (version 16.114; Microsoft Corp., Redmond, WA, USA) (see Appendices).

Compliance was assessed against the East Cheshire NHS Trust Hypoglycaemia Management Guidance, which is based upon those set out by NICE guidance and JBDS. Local trust guidance was found on the Trust Intranet by searching for "Hypoglycaemia Management Guidance". We used key components of local guidance - such as consciousness level or seizures, glucose concentration, and the 15-minute monitoring time - which determine the treatment pathway, as criteria to assess compliance. The trust's hypoglycaemia management algorithm is presented in Figure 1.

**Figure 1:**
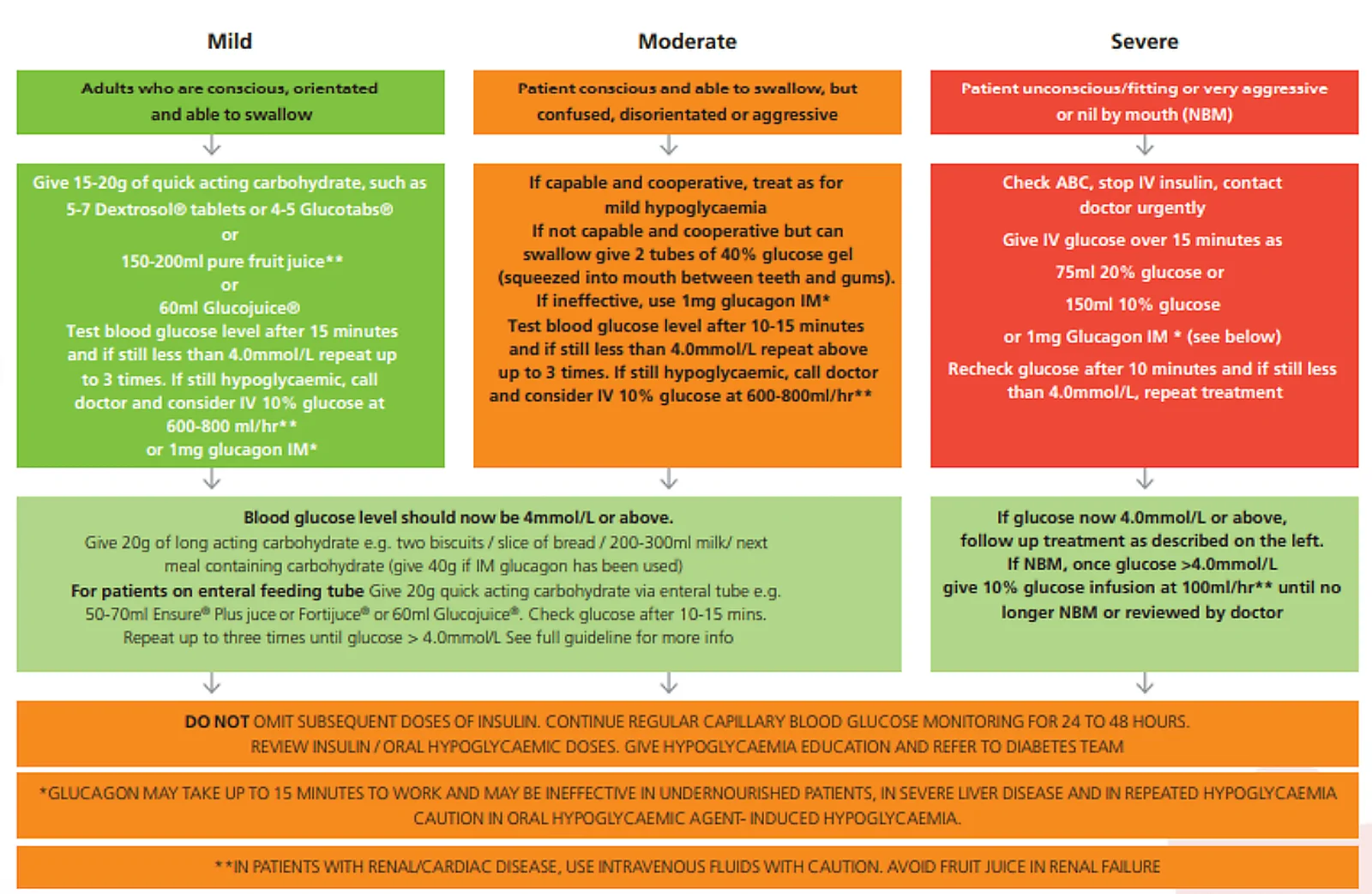
Local trust guidance for hypoglycaemia management IV: intravenous; IM: intramuscular; ABC: Airway, Breathing and Circulation; g: grams; mL: millilitres

No sample size calculation was undertaken and was limited by the low incidence of inpatient hypoglycaemia within the available time frame. This was in consideration of the time-intensive nature of case identification and evaluation needed.

Setting

This QIP took place in Macclesfield General District Hospital (MGDH), Cheshire, in the United Kingdom. MGDH is a small- to moderate-sized acute care hospital, with several acute and non-acute medical wards.

Statistical analysis

All outcomes were analysed as categorical variables; given the small sample size and the presence of independent variables, Fisher's exact test was undertaken to measure statistical significance. As per trust guidance, all cases should be monitored within 15 minutes; therefore, the two groups were compared as within 15 minutes or above 15 minutes. The correct treatment pathway was guided by the documented state of the patient and their respective glucose level. For statistical analysis, patient status examination was split into: stated or not stated.

When performing a Fisher's exact test for the primary outcome, cases with no documentation were excluded, as we cannot assess the patient's accurate well-being. Therefore, we cannot assess if the treatment pathway chosen was correct. Fisher's exact tests were performed using the Free Online 2×2 P-Value & Odds Ratio - Stats Unlock Online calculator (StatsUnlock, USA) [14].

Glucose monitoring

In line with local trust and NICE guidance for this study, a hypoglycaemia episode was classed as <4 mmol/L. Two categories were defined: mild or severe. A severe hypoglycaemic episode was defined as less than 3 mmol/L. Symptoms of hypoglycaemia were not used to define severity category as patient well-being documentation was not always fully present. A resolution of hypoglycaemia episodes was defined on an individual case basis; however, a patient with a glucose of greater than 4 mmol/L upon re-monitoring was defined as resolved. Glucose monitoring times were also put into categories of less than 15 minutes, 30 minutes, 45 minutes, and 60 minutes or greater in order to simplify interpretation. Capillary glucose readings only were collected by nursing staff using a 4SURE Smart DUO Meter Kit manufactured by Nipro Corporation (Osaka, Japan). Continuous glucose monitoring was not used, and a confirmatory plasma glucose sample was not requested from the laboratory.

Patient documentation

Patient-specific well-being status variance was classified by consciousness or confusion, nil by mouth, presence of seizure, ability to swallow, or use of enteral feed (Table 1). These parameters match those suggested by trust guidance as measures of severity.

**Table 1:** Description of patient well-being within nursing documentation

Patient State	Description
A	Conscious, oriented and able to swallow
B	Conscious, but confused and disoriented
C	Unconscious and/or seizures
D	Nil by mouth
E	Enteral Feed

Patient documentation was separately classed as complete, partial, incomplete or not documented. The fully documented category required the inclusion of the patient status, the glucose concentration, a nurse signature on the final document and the management path used. A loss of one aspect of these would classify a partial document. Unsigned documents were classed as incomplete. This was also a subjective assessment, which aimed to reflect the general impression of documentation standards.

Management of hypoglycemia

Treatment pathway was classed into one of three categories, A to C, representing the range of treatment appropriate for the severity of the hypoglycemia (Table 2).

**Table 2:** Range of treatment choices available to medical and nursing teams mL: millitre; IM: intramuscular; IV: intravenous

Route A	Route B	Route C
GlucoJuice 60 mL	Buccal Glucogel/dextrogel	1 mg Glucagon IM if no IV access
4x GlucoTabs	Repeat route A three times	150 mL-200 mL 10% IV glucose
150-200 mL fruit juice	Or IM Glucagon	-
Three to four teaspoons of sugar in water	-	-
Dextrosol tablets	-	-

Quality standards

Local hypoglycaemia guidance was held as the standard operating procedure.

Nursing interventions

Teaching and Feedback

On 14 May and 10 June 2026, before the commencement of the post-intervention group assessment dates, one-to-one teaching and feedback were delivered to nursing staff present and available on all medical wards. Teaching involved highlighting key sections of local guidance protocols such as documenting patient well-being, glucose monitoring times and suggested treatment pathways. During feedback or shortly afterwards, feedback was taken from nursing to discern challenging areas of hypoglycaemia management for staff and to assess confidence. Eleven nurses provided feedback via an anonymised current web version of Google Sheets (Google LLC, Mountain View, CA, USA; June 2026) made via Google Drive (Google LLC), which was accessible via a quick response (QR) code provided or an online link sent by email, which could be used after the teaching. Answers were taken on a 5-point scale of their confidence or as open text when asked their personal thoughts on the matter. Eleven nurses (55%) completed the feedback questionnaire out of the 20 who attended. All free text responses were read and grouped into three main areas according to recurring themes: workload or staffing, documentation and knowledge. Two nurses did not fill out the free text box.

During visits to each medical ward, local guidance protocols, including flowcharts and management criteria leaflets, were also posted within drug store rooms next to "hypo boxes" and in ward offices to act as prompts.

This QIP was conducted as a local clinical practice evaluation and therefore was not subject to formal NHS Research Ethics Committee approval and was registered with the Trust Clinical Audit Effectiveness Team (audit number 3947).

## Results

Teaching intervention feedback results

After teaching, there was an increase from 73% (n=8) to 91% (n=10) in the maximum scaled (5 out of 5) confidence in hypoglycaemia management. This is shown in Figures 2-3. All nurses (n=11) who gave feedback understood, after teaching, where to find the ward hypoglycaemia kit, also termed "hypoboxes", local trust guidance and were aware that the baseline minimum time to repeat a glucose level was <15 minutes for all patients. Figure 4 also represents the open text responses given when asked what challenges nurses face in hypoglycaemia management.

**Figure 2:**
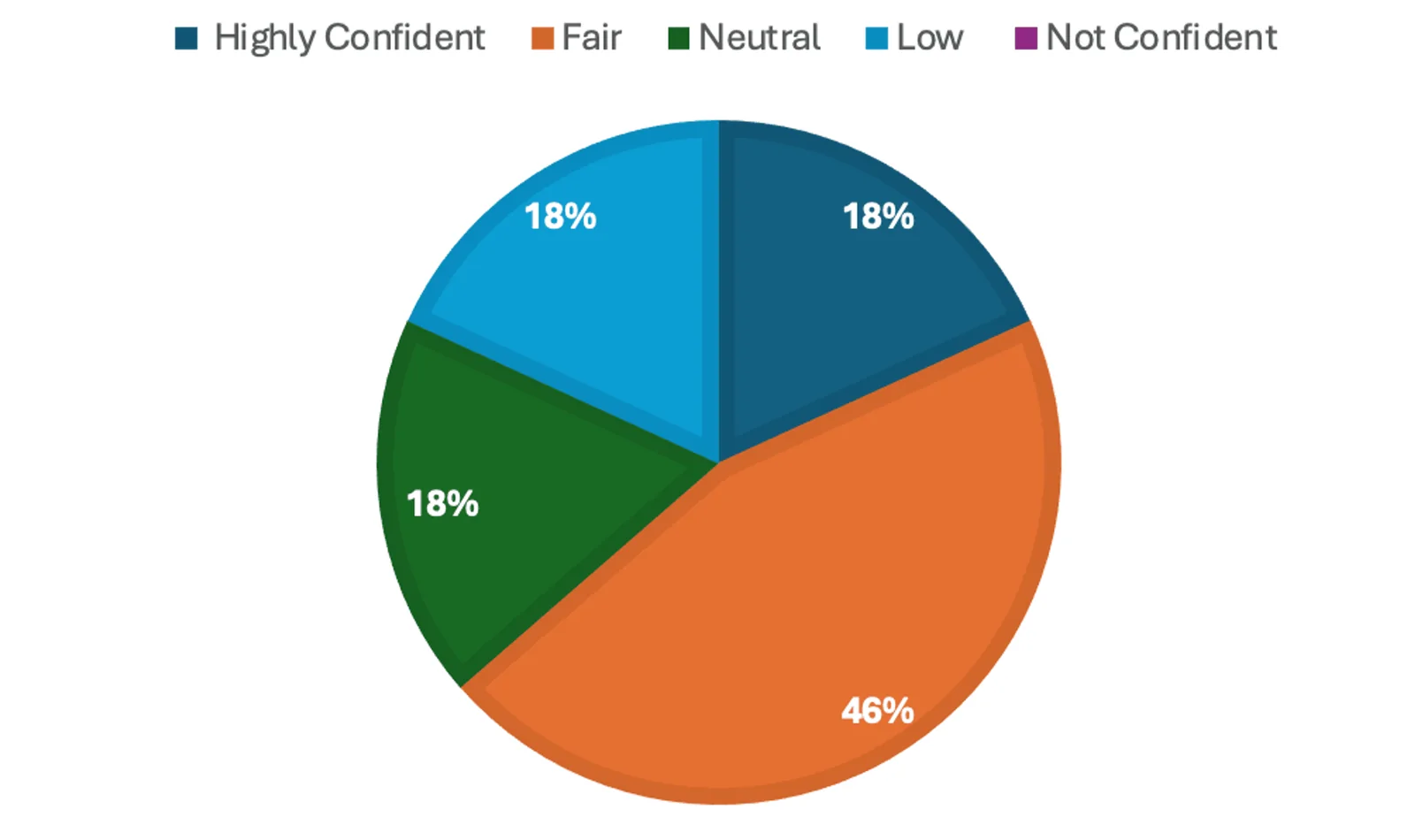
Before teaching, how would you rate your confidence in identifying, managing and escalating the management of hypoglycaemia in patients?

**Figure 3:**
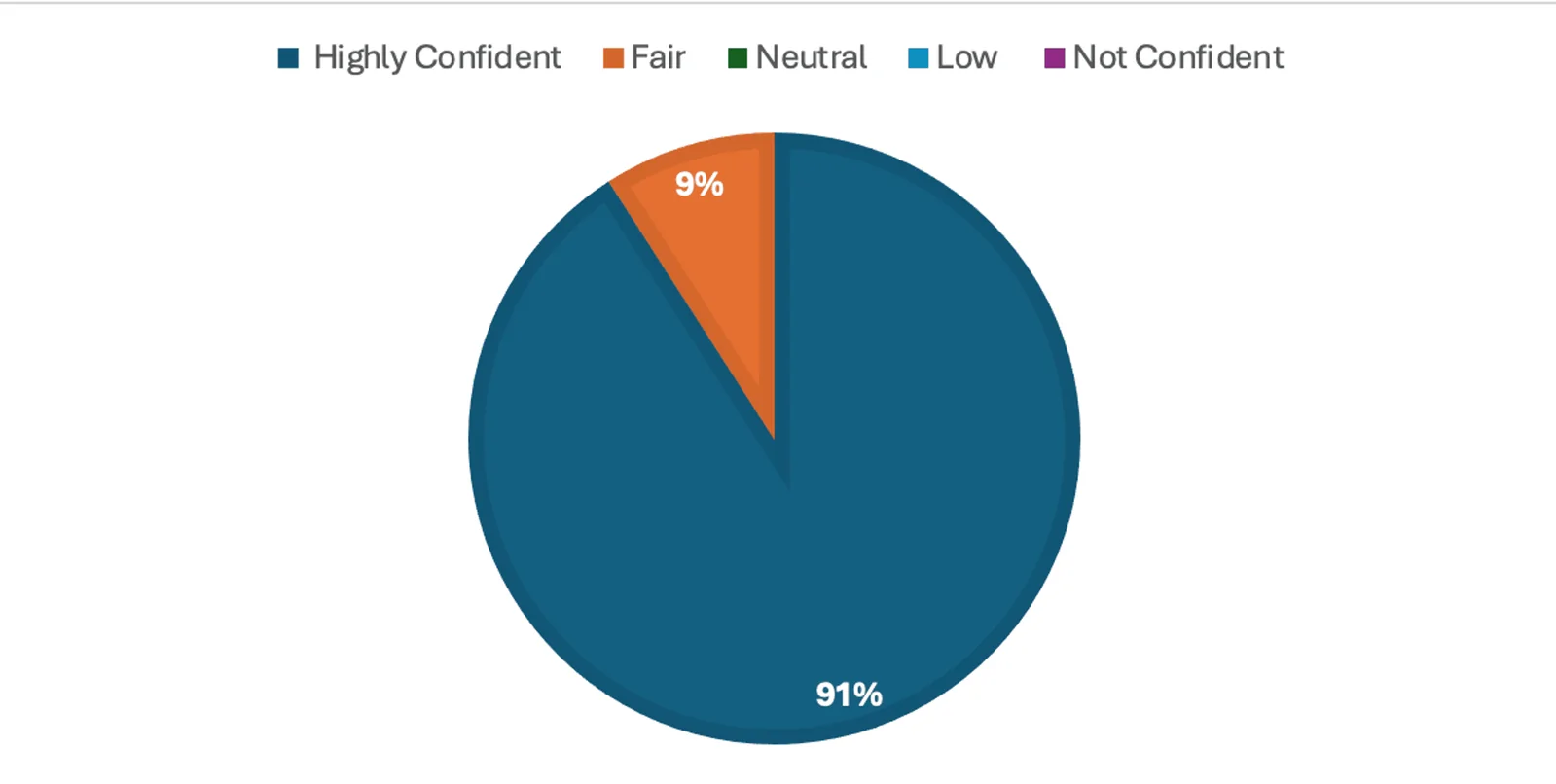
After teaching, how is your confidence in identifying, managing and escalating the management of hypoglycaemia in patients?

**Figure 4:**
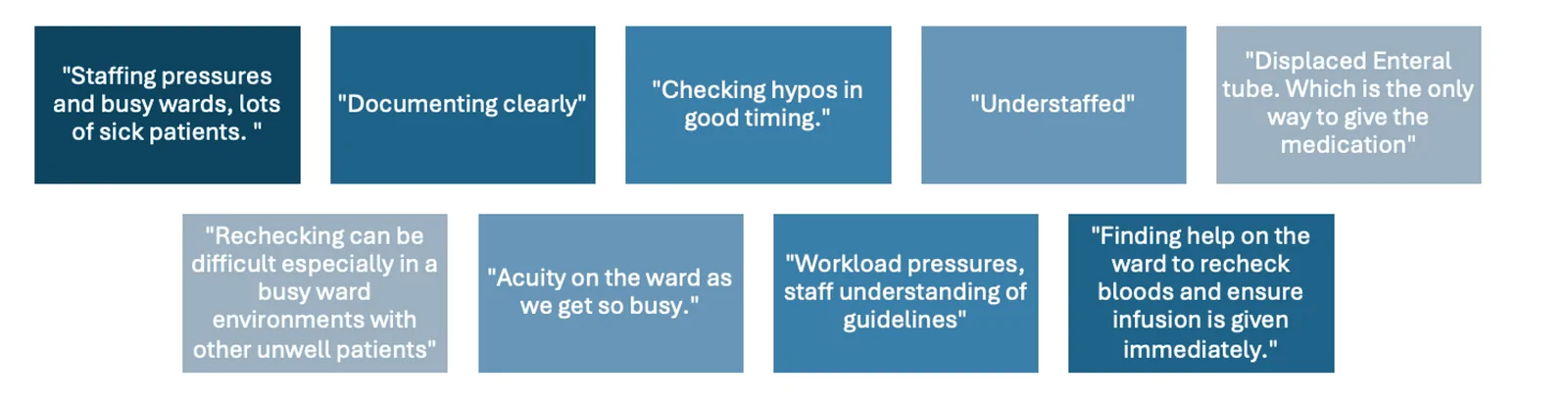
Response to nursing challenges: what do you feel is the biggest challenge to treating hypoglycaemia quickly, rechecking blood glucose levels and documenting?

Cohort characteristics

Baseline and post-intervention cohorts included 20 cases of hypoglycaemia each (n=20). These cases consisted of 13 patients in the baseline group and six patients in the later group. Patients within the baseline study aged from 30 to 91 years, with an average of 68.5 years and a median of 79 years. In comparison, the post-intervention group patients’ ages ranged from 68 to 90 years, with an average of 78.6 years and a median of 76.5 years. In the baseline study, mild hypoglycaemia was most common at 60% (n=12), and 40% (n=8) had severe hypoglycaemia. Severe hypoglycaemia (<3.0 mmol/L) episodes were identified in 40% (n=8) in both groups.

When comparing regular anti-diabetic medication regimens (Figure 5), insulin treatment was most common in both groups, at 40% (n=8) in the baseline group compared to 60% (n=12) in the post-intervention group. Oral anti-diabetic medication alone was used in 30% (n=6) in the baseline group, and no patients used oral anti-diabetic medication alone in the post-intervention group. Some patients used a mix of both regimens in each study; for example, 10% (n=2) in the baseline group and 35% (n=7) in the post-intervention group. A smaller proportion of patients did not use anti-diabetic medication regularly: 15% (n=3) in the baseline group and 5% (n=1) in the post-intervention group.

**Figure 5:**
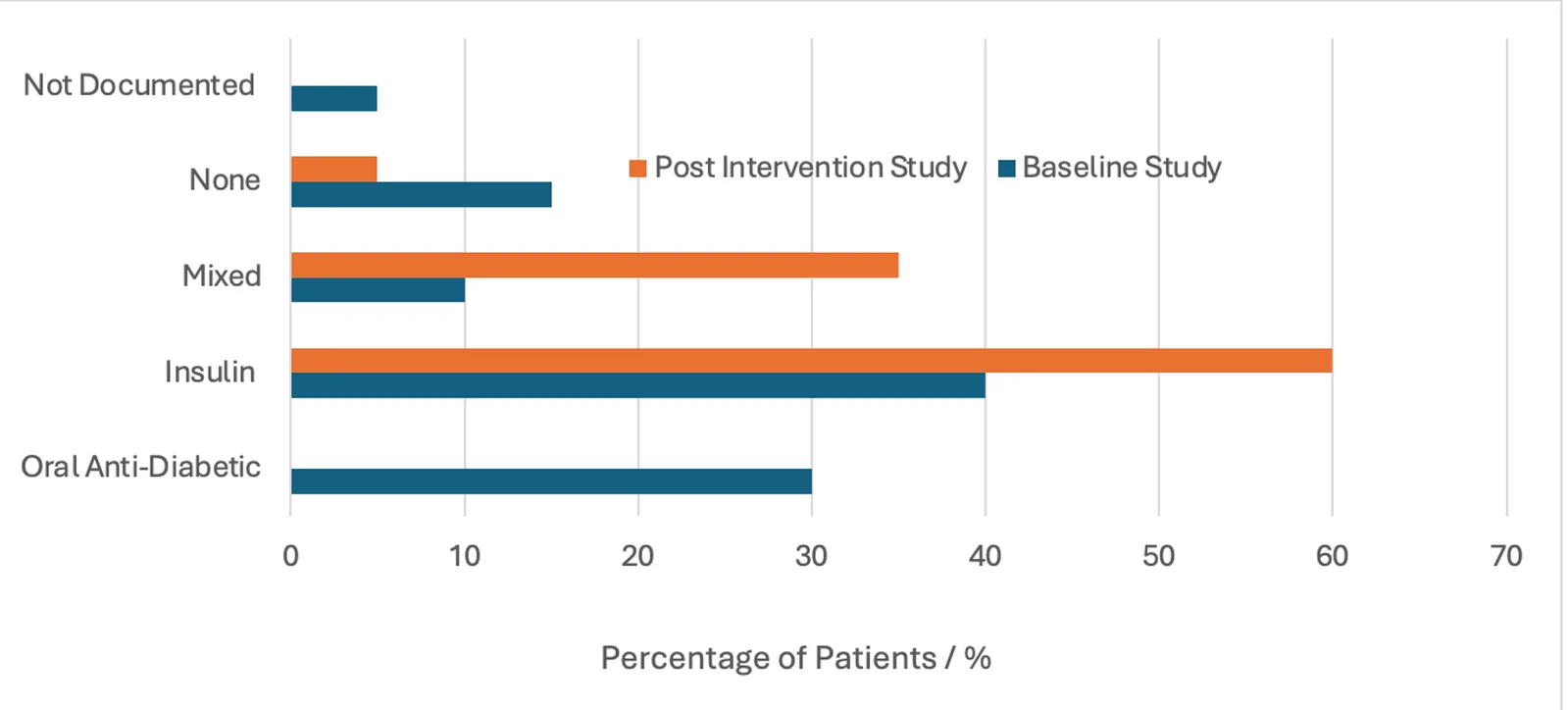
A variety of anti-diabetic medications used regularly by patients

In the baseline study, 10% (n=2) did not have a diagnosis of any formal diabetes diagnosis. In the post-intervention group, 5% (n=1) did not have a formal diagnosis. In the baseline study, one end-of-life (EOL) patient accounted for four cases. This is illustrated in Figure 6. In the post-intervention group, 95% (n=19) of cases involved a patient with type 2 diabetes mellitus, compared to 70% (n=14) in the baseline group. In the prevalence of type 2 diabetes in baseline versus post-intervention, no statistically significant difference was identified (p=0.09, 95% CI 0.878-75.479, OR 8.143, φ=0.329).

**Figure 6:**
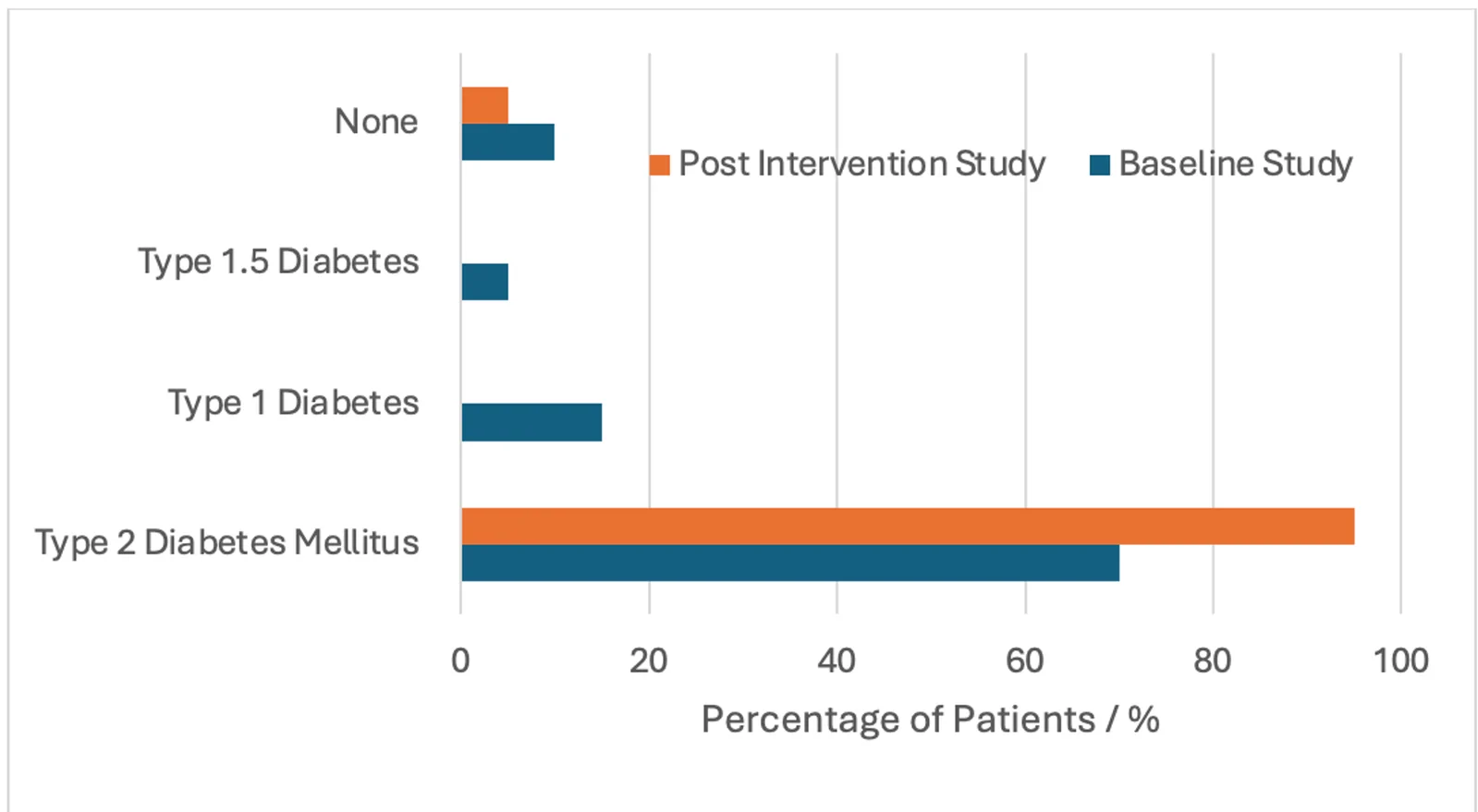
Prevalence of diabetes type diagnosis present within medical inpatients

Patient documentation

General nursing documentation completeness worsened by 10% (n=2), and in the post-intervention group, the number of nursing documents not explicitly stating patient status remained constant at 30% (n=6). For example, in the baseline audit, 45% (n=9) of general nursing documentation was deemed fully complete, 45% (n=9) was partial, and 10% (n=2) had no nursing documentation. Post-intervention, the degree of full general nursing documentation dropped to 35% (n=7), and 45% (n=9) remained at a partial documentation ranking. There was a rise to 20% (n=4) in cases with no general documentation. This is illustrated in Figure 7.

**Figure 7:**
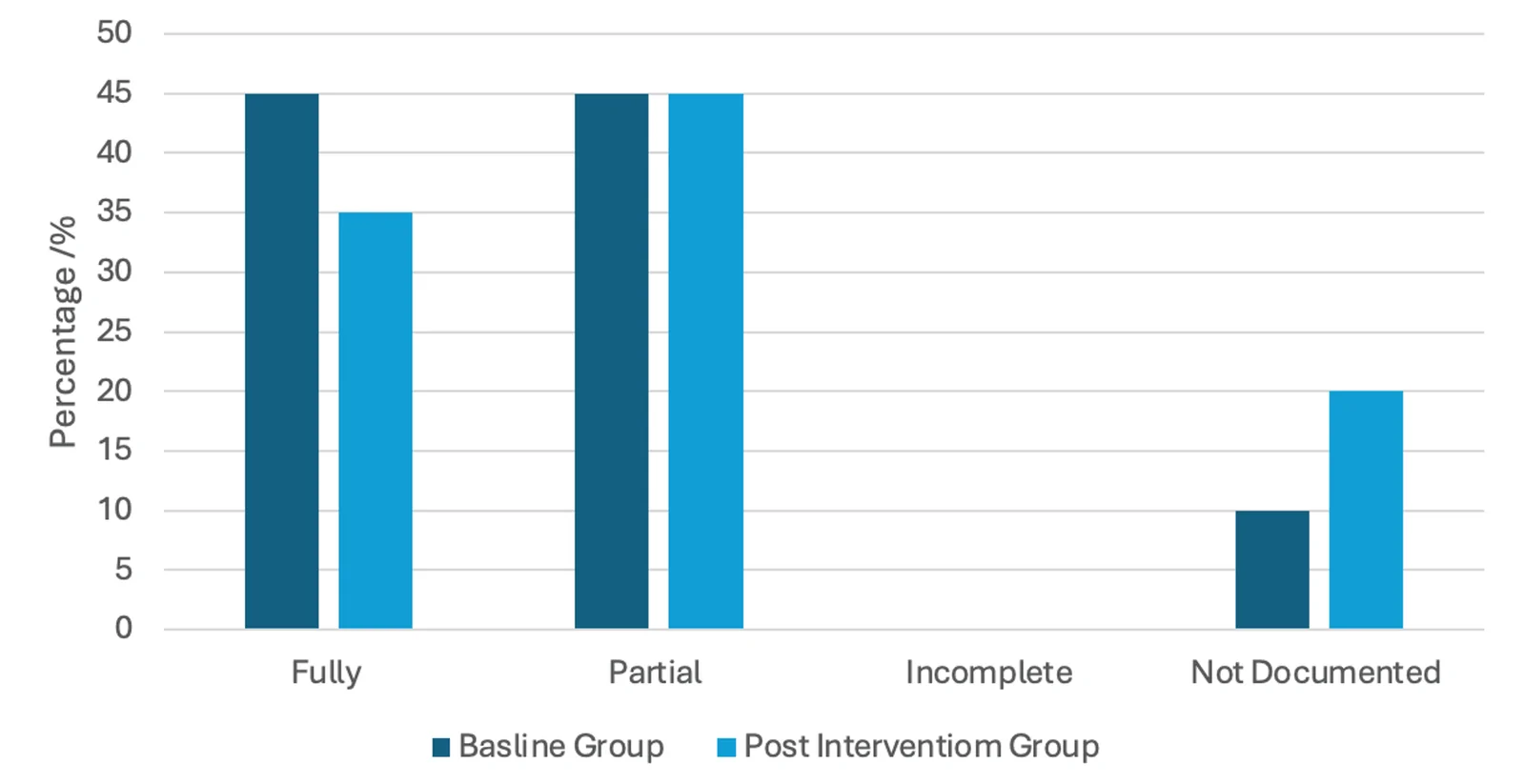
Assessment of patient's general nursing documentation with hypoglycaemia

Nursing documentation of specific patient well-being status during a hypoglycaemia episode was evaluated. It was found that 65% (n=13) of patients were classed as status A (alert). Patients with status B were present in 5% (n=1), and the patients’ well-being was not documented in 30% (n=6) of cases. There was no documentation of specific patients’ well-being status post-intervention in 30% (n=6). In the post-intervention group, 70% (n=14) were classed as status A. This is illustrated in Figure 8. There was no statistical difference in the post-intervention and baseline groups when comparing stated and not stated patient status (OR 1.00, 95% CI 0.26-3.87; p=1.000, φ=0.0).

**Figure 8:**
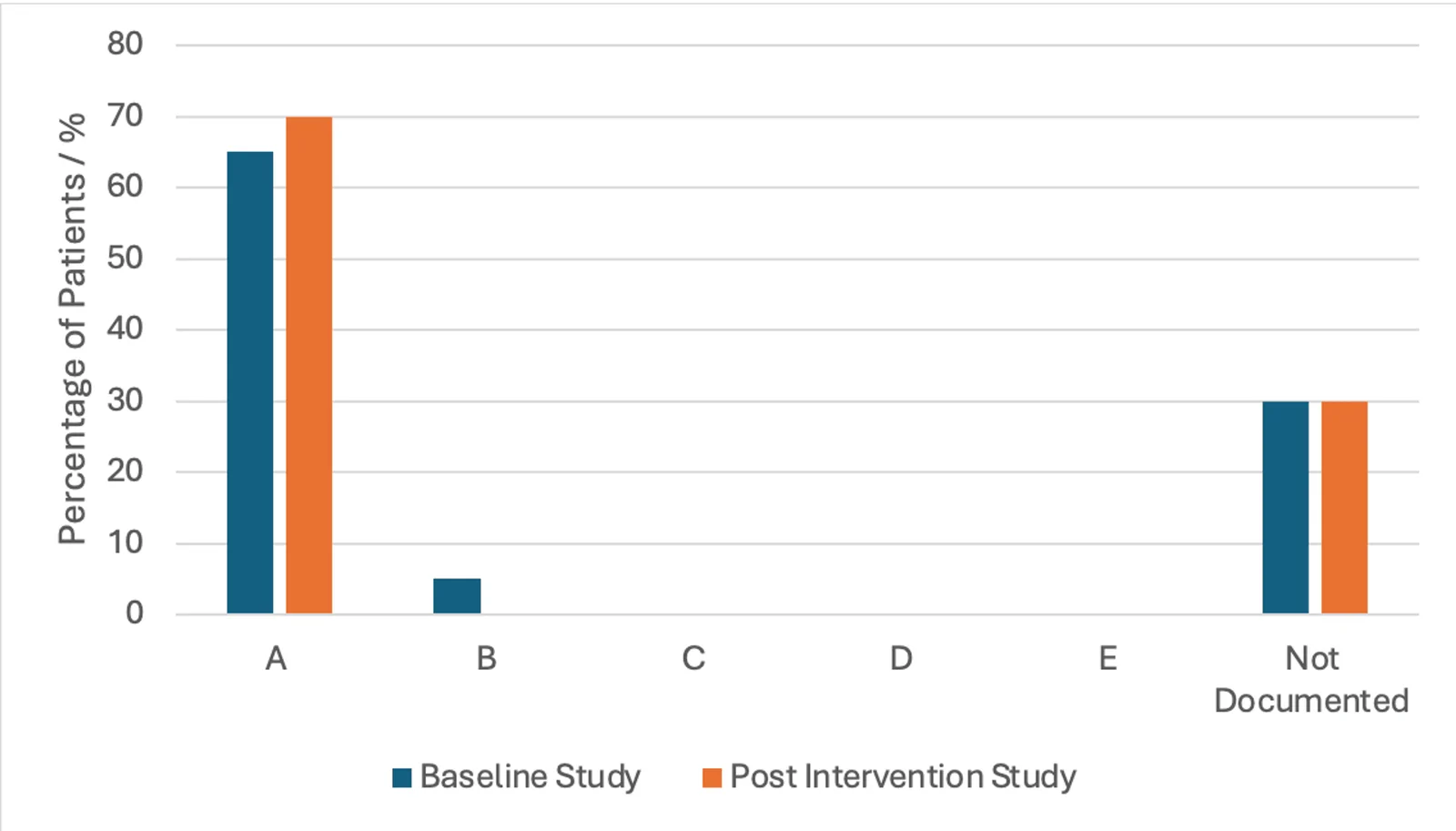
Patient status level documented by nursing staff

Glucose monitoring

In the baseline audit, repeat glucose readings were most commonly documented in the over one hour plus category, 30% (n=6). The second most common time interval in the baseline group was 15 minutes at 25% (n=5). The 30-minute and 45-minute brackets accounted for 20% (n=4) and 15% (n=3) in the baseline group. In the baseline study, 5% (n=1) was not documented. In one instance, a patient refused monitoring, and in another, no repeat sample was taken at all. Post-intervention, the most common time interval was one hour or greater at 30% (n=6). However, there was an increase from 15% (n=3) to 30% (n=6) in the proportion of glucose monitoring taken at 45 minutes in the post-intervention group. It was less likely for monitoring to take place at shorter time intervals; for instance, 25% (n=5) were within 30 minutes, only 5% (n=1) were taken within 15 minutes, and 5% (n=1) were not documented in the post-intervention group. In each group, one patient refused further monitoring of blood glucose levels (5%; n=1). This is demonstrated in Figure 9. Although there was a decrease in the proportion taken at 15 minutes, these results did not reach statistical significance (OR 0.18, 95% CI 0.02-1.81; p=0.167, φ=0.264).

**Figure 9:**
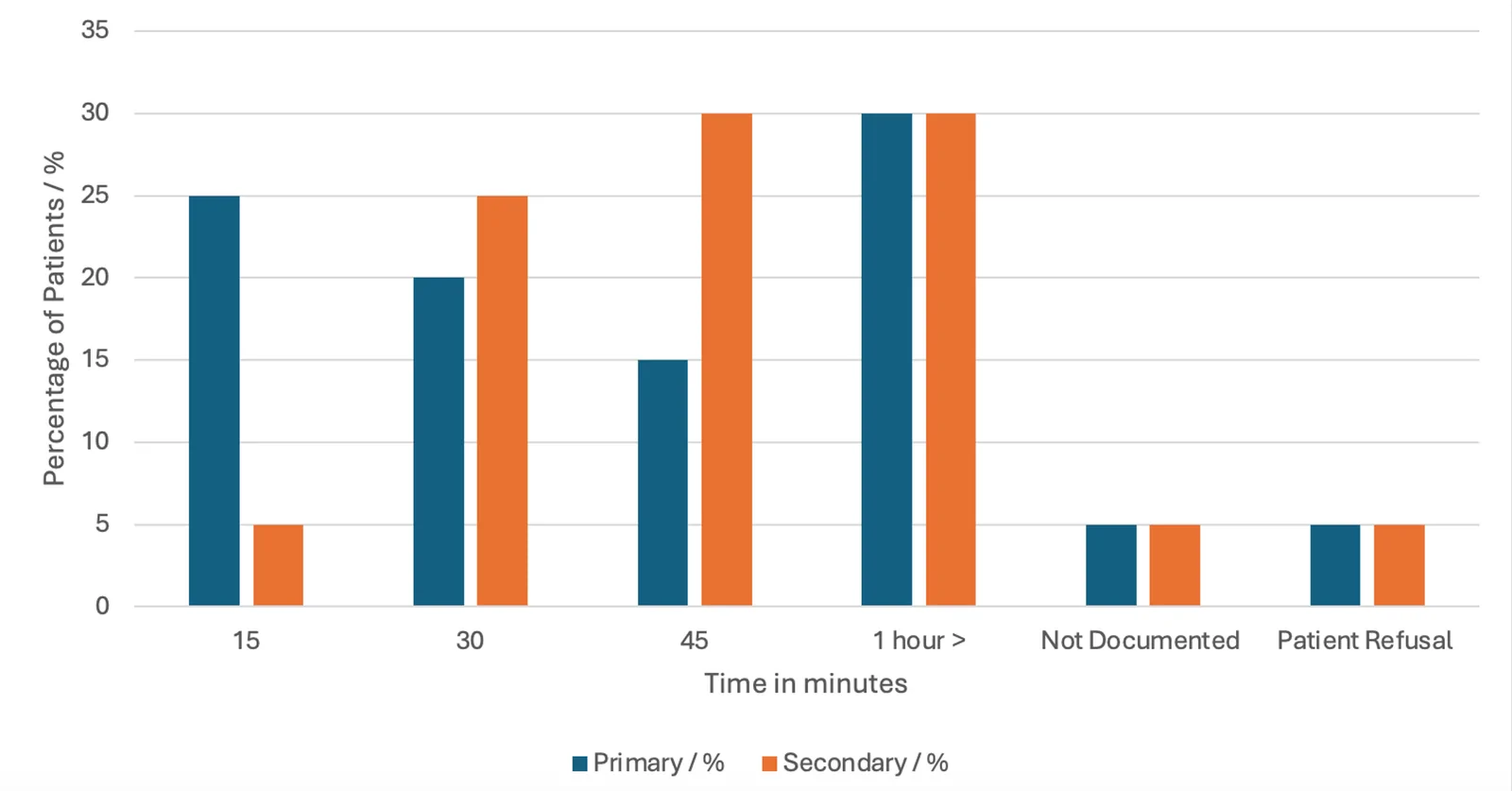
Time interval to monitor capillary glucose by nurses after hypoglycaemia is identified

Management route used for hypoglycemia

In the primary audit, 40% (n=8) used route A, 10% (n=2) used route B, 30% (n=6) used route C, 5% (n=1) received no explicit treatment, and 15% (n=3) had no documentation. Post-intervention, 80% (n=16) used route A management, and 5% (n=1) used route C. In no cases was route B used, and in n=3, no treatment was used. This is shown in Figure 10. There was a statistically significant increase in post-intervention (OR 11.14, 95% CI 1.11-112.01; p=0.033) with an effect size of 0.445 in the use of route A patients.

**Figure 10:**
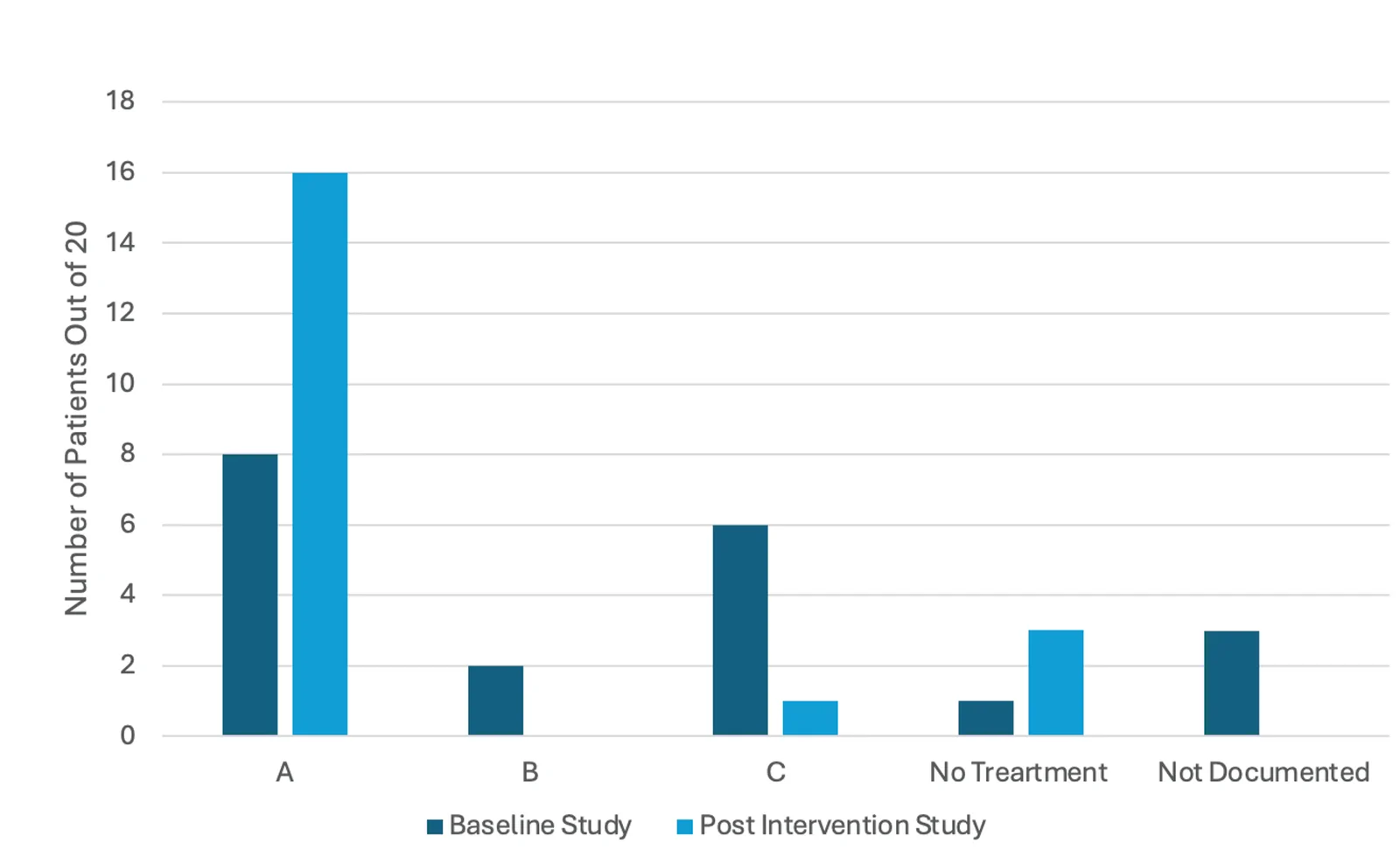
Choice of hypoglycaemia treatment used

Escalation and prevention planning

In the baseline audit, 40% (n=8) had severe hypoglycaemia, and 65% (n=13) were patients in status A; the doctors were directly informed in 50% (n=10) of cases. In response, 40% (n=8) action plans were made to reduce the risk of hypoglycaemia, such as reducing insulin or anti-diabetic medication doses. In the post-intervention group, again, 40% (n=8) had severe hypoglycaemia, and the doctor was informed in 15% (n=3) of occasions. In response, a doctor made a prevention plan in 30% (n=6) of these cases.

## Discussion

Baseline and post-intervention groups were comparable in median age and identical in prevalence of mild hypoglycaemia and severe hypoglycaemia episodes. The post-intervention group was type 2 diabetes mellitus dominant (n=19 versus n=14); however, this did not reach statistical significance (p=0.09,95% CI 75.479, OR 8.143, ϕ=0.329). Insulin-only use was 20% (n=4) more common in the post-intervention group, and there was 25% (n=5) greater use of a mix of both regimens. Moreover, the post-intervention group had six patients, indicating a clustering of observations, which would reduce the independence of observations and reduce the effective sample size. According to a recent systematic review by Li et al., these factors should be taken into account; however, clinically, as described, the post-intervention groups could have represented a higher-risk group due to higher use of insulin use, for example [15].

Fully completed general nursing documentation was low in both groups at 45% (n=9) and 35% (n=7), respectively. The rate of missing general nursing documentation increased from 10% (n=2) to 20% (n=4) post-intervention. Moreover, when assessing whether nurses stated the patient's status and well-being, this remained at 30% (n=6) in both groups; therefore, there was no statistically significant difference (p=1.00). In line with NHS quality improvement methodology and nurses' teaching methodology, future cycles should repeat teaching regularly to aid in consolidating knowledge and improve confidence [16,17].

In the baseline study, 65% (n=13) of patients were status A (alert, conscious, or able to swallow); however, route A management was used in only 40% (n=8) of patients. Post-intervention, there was an increase of 40% (n=8) in the use of route A management; this is more likely representative of the correct treatment response, as 70% (n=14) of patients were classed as status A. Post-intervention, there was statistically significant improvement in the correct treatment pathway used overall, which included those with non-status A well-being (p=0.033). Therefore, the primary outcome was in fact achieved by definition. However, this must take into account that the underlying population size was small, and the statistical analysis has a wide confidence interval. This may indicate uncertainty about whether results are due to the intervention used.

However, upon further inspection, the baseline group possessed a patient on EOL care, which could have increased the use of route C, for instance, if the patient was not able to eat. Nevertheless, this patient could be someone who should not have had monitoring if commenced on EOL care. Several cases were excluded from statistical analysis as their well-being could not accurately be determined; therefore, the observations may not be representative of all hypoglycaemia episodes observed and could introduce selection bias.

The time interval to monitor glucose showed the most non-compliance with guidance. All patients are expected to be rechecked in 15 minutes. In the baseline group, according to documentation, only 25% (n=5) of patients achieved the 15-minute window; this dropped to just 5% (n=1) post-intervention. The variance in 15-minute monitoring between groups did not reach statistical significance (p=0.167). Therefore, there was no statistically significant improvement in this secondary outcome. In the baseline group, glucose monitoring occurring at 45 minutes and over one hour was 15% (n=3) and 30% (n=6), respectively; this increased to 30% (n=6) in each bracket in the post-intervention group. Overall, this indicated a worsening in glucose monitoring post-intervention. This result is in contrast to the choice of hypoglycaemic treatment used, which had improved.

The underlying method of monitoring glucose includes the use of a Nipro 4 Sure Smart glucose meter (ForaCare Suisse AG, St. Gallen, Switzerland), which nursing staff must manually record the glucose concentration onto DCS. Therefore, in practice, it is possible that the documented time did not represent the accurate capillary sample time. Therefore, this could have introduced an element of recording bias.

When escalating to a doctor, direct discussion with doctors decreased by 35% (n=7) in the post-intervention group (n=3). Both groups had 40% (n=8) severe hypoglycaemia, of whom 65% (n=13) and 70% (n=14) were stated as status A, respectively. Local trust guidance suggests escalating in severe hypoglycaemia in patients who cannot swallow, have failed route A management or are unconscious, for instance. When including only fully documented cases, no patients had reduced consciousness or had seizures. Therefore, it could be expected that the proportion escalating to a doctor would be lower. The 35% (n=7) reduction could also have been due to the lower patient number in the later study. In this study, a number of factors were not measured, such as patients' comorbidities and existing individual treatment plans and glucose targets, symptom burden or previous length of stay. Consequently, these could act as confounding variables. In addition, because of this, a choice to escalate could not solely be judged in this study; however, it may reflect the variance in patients between studies by chance.

The teaching intervention increased confidence in almost all nurses and aided in improving awareness. Although trust guidance was posted in all medical wards, 11 out of 20 (55%) nurses likely represented a small proportion of total nursing staff. Future cycles should include more staff in order to have a greater effect on outcomes. As shown by the nursing feedback, there remained other uncontrolled barriers. For example, a recurring theme was reported in the workload or staffing category. Workload pressure could act as another confounding variable; a recent systematic review suggests that nurses under workload pressure have significant potential to miss patients' care needs [18]. A further measure to help this could involve the implementation of a digital template used on DCS, which will prompt nursing staff to recheck glucose levels and ensure patient well-being checks are documented automatically. This could reduce the time taken to document and could offer direct links or prompts to local guidance pathways, for instance.

Limitations

There are several limitations identified within this study. The sampling method used patients from the medical wards only, which reduced the representative picture of hypoglycaemia treatment in the whole hospital and introduced selection bias. Moreover, both studies used 20 cases, and a relatively low number of patients came from each group. This resulted in a wider confidence interval and limited precision. Multiple hypoglycaemic episodes resulted from fewer patients, especially in the post-intervention group; therefore, a clustering effect was observed and reduced the likelihood of independent results. The study design could have been improved further by including patients’ specific medication and dose, which was not taken into account. Moreover, only cases of <4 mmol/L were included; some symptoms of hypoglycaemia and initiation of management can occur above this level due to relative hypoglycaemia [19]. Identifying cases based upon a biochemical threshold rather than a clinical symptom or presentation could have introduced selection bias. Moreover, in future cycles both aspects could be used as search tools in order to select a cohort. It is also recognised that this study did not use confirmatory plasma glucose concentrations, and patients with continuous blood glucose monitoring were not measured.

As shown by the study by Pratiwi et al. [7], several variables can affect the risk of hypoglycaemia occurrence, such as sepsis, specific medications, kidney function, anaemia, hormone deficiency and low body mass index. These could have acted as unmeasured confounding variables and affected the observed results.

During the intervention, 55% (n=11) of nurses filled out the feedback, which could have introduced non-response bias in the confidence improvement result and therefore overestimated the effect of the teaching. Future cycles could use anonymised paper feedback responses to minimise technological barriers, for example.

## Conclusions

This QIP used in-person teaching, poster prompts and feedback interventions in order to improve compliance with hypoglycaemia management and documentation among nursing staff. In the post-intervention group, there was an observed increase in compliance with the suggested hypoglycaemia management pathways. However, compliance was not 100% in any primary outcome. Moreover, due to suboptimal documentation and exclusion of such cases from analysis, observations may not be fully representative. Secondary outcomes were not achieved in the areas of patient status documentation or timely monitoring. This study may suggest that further cycles are warranted, especially in the area of glucose monitoring intervals, which showed the lowest degree of compliance, with a focus on nursing documentation.

## Figures and Tables

**Figure 11:**
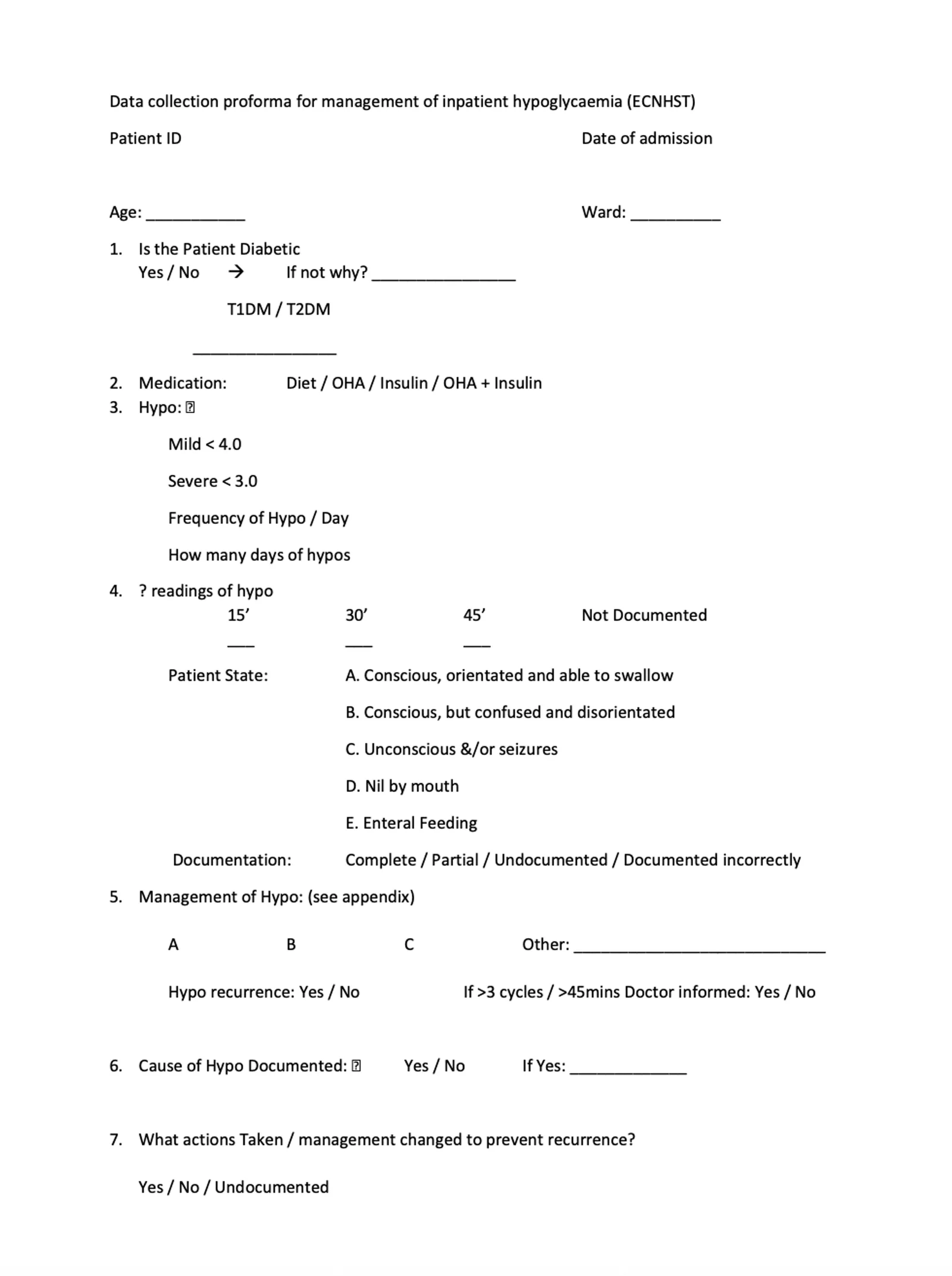
Standardised hypoglycaemia audit proforma used to collect data Proforma collects several data points, including glucose readings, patient status, documentation completeness and presence, action plan, escalation and recurrence frequency.
